# Detection of Multiple Cracks in Four-Point Bending Tests Using the Coda Wave Interferometry Method

**DOI:** 10.3390/s20071986

**Published:** 2020-04-02

**Authors:** Xin Wang, Joyraj Chakraborty, Antoine Bassil, Ernst Niederleithinger

**Affiliations:** 1 Bundesanstalt für Materialforschung und-prüfung (BAM), Unter den Eichen 87, 12205 Berlin, Germany; ernst.niederleithinger@bam.de; 2 Research and development department, NeoStrain Sp. z o.o, Lipowa 3, 30-702 Krakow, Poland; joyraj@neostrain.pl; 3 IFSTTAR, COSYS-SII, Route de Bouaye, F-44344 Bouguenais, France

**Keywords:** coda wave interferometry, reinforced concrete, cracks, SHM, damage detection

## Abstract

The enlargement of the cracks outside the permitted dimension is one of the main causes for the reduction of service life of Reinforced Concrete (RC) structures. Cracks can develop due to many causes such as dynamic or static load. When tensile stress exceeds the tensile strength of RC, cracks appear. Traditional techniques have limitations in early stage damage detection and localisation, especially on large-scale structures. The ultrasonic Coda Wave Interferometry (CWI) method using diffuse waves is one of the most promising methods to detect subtle changes in heterogeneous materials, such as concrete. In this paper, the assessment of the CWI method applied for multiple cracks opening detection on two specimens based on four-point bending test is presented. Both beams were monitored using a limited number of embedded Ultrasonic (US) transducers as well as other transducers and techniques (e.g., Digital Image Correlation (DIC), LVDT sensors, strain gauges, and Fiber Optics Sensor (FOS)). Results show that strain change and crack formation are successfully and efficiently detected by CWI method even earlier than by the other techniques. The CWI technique using embedded US transducers is undoubtedly a feasible, efficient, and promising method for long-term monitoring on real infrastructure.

## 1. Introduction

Infrastructure is the foundation for economic development of the society of a country. Along with population growth, a rising rate of urbanization, and more communication between different regions, the demand for more infrastructure increases (i.e., housing, bridges, highways, etc.). RC is the most widely used material in the construction industry due to its durability, rapid construction, and low maintenance cost. The combination gives the advantage of using two materials as a composite material (RC); concrete has a strong compressive strength and steel has a high tensile strength. This allows almost unlimited range of uses of RC in infrastructure [1]. Usually, reinforced concrete structures are designed for a service life of more than 50 years. However, mechanical and environmental factors or even excessive use accelerate the deterioration of concrete structures. When damage in the structure reaches a certain level, the structure could collapse if necessary maintenance is not carried out in time. Most degradation and failure mechanisms are associated with the enlargement of cracks. Thus, evaluating the health condition of a structure during its service life is necessary.

Structural Health monitoring (SHM) is playing an increasing role in infrastructure management. SHM refers to permanent monitoring using installed sensors, including processing and interpretation in terms of structure health. The Non-Destructive Testing (NDT) technique is the highest quality assurance tool which allows the assessment of quality and condition of structure with out damaging the original structure. A wide variety of NDT methods have been developed and used for decades in the manufacturing industry and in the civil engineering domain, for example, ground penetrating radar [2,3,4], laser testing method [5,6], DIC [7,8,9] and FOS [10,11], etc. Among these methods, many of them can be used for long-term SHM.

The sonic method is one of the most reliable and commonly used methods among all NDT techniques because of the direct relationship of wave velocity and the physical and mechanical properties of the material. Acoustic Emission (AE) is an efficient passive method to detect impending damage in concrete by analysing the wave generated by a sudden release of energy from the material under investigation. The application of AE used in infrastructure is described in [12,13,14]. However, AE signals are usually weak and strongly disturbed by the noise occurring in field applications making the analysis of signals more complicated. The cost of AE devices is also high. The Ultrasonic Pulse Velocity (UPV) test is the most simple and classic method used to evaluate the quality of concrete by measuring the propagation time of waves. Higher velocity indicates better quality. The compressive strength of the concrete can be estimated using UPV [15]. However, when the change in the medium is relatively small, the UPV method is not sensitive enough. A new method, Coda Wave Interferomertry (CWI), using scattered waves to detect small changes in the medium, has been developed [16]. Existing studies have shown the high sensitivity of CWI to detect temperature change [17,18,19], stress variation [20,21] and damage [22,23] in small, laboratory size specimens. The CWI method has been also applied to large-scale specimens in outdoor environments [24] and successfully implemented on real structures [25,26,27]. Traditional NDT tools are normally used on the surface of the structure with limited penetration depth. To focus more on the changes inside the structure, a new embedded US transducer was developed [28]. Early stage single crack detection was studied by combining CWI using the new embedded US transducers and FOS methods, see details in [29]. The more complicated case for multiple cracks has not yet been thoroughly analysed. In this paper, studies of the occurrence of multiple cracks in RC specimens during four-point bending tests monitored by a combination of CWI, FOS, DIC as well as other classic sensors are presented.

The paper is organised starting with the theory of the CWI method presented in Section 2. Experimental investigations, including the transducer location, data acquisition system, and specimen parameters, are described in detail in Section 3. Section 4 introduces the crack positions, deflection of two beams, crack opening moment detected by FOS and DIC and the result of the CWI method. A comparison of all the sensors and techniques is discussed in Section 5. The paper is concluded in Section 6.

## 2. Coda Wave Interferometry (CWI) Technique

**Figure 1:**
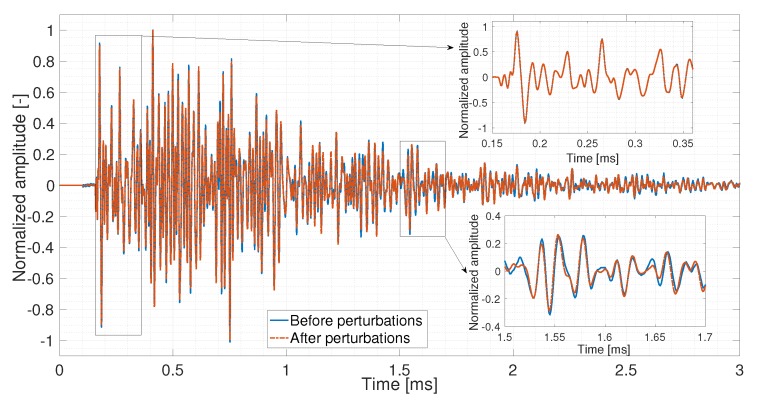
Signals recorded before and after small perturbations in the medium.

Traditionally, sonic methods or vibration measurements for Structural Health Monitoring (SHM) are performed below 20 kHz; the wavelength is larger than or of the order of the macroscopic size of the structure [30]. Thus, it has a limitation in detecting the microscopic features and many typical defects. However, when the working frequency range exceeds 50 kHz, the wavelength is in the order of the size of the aggregates, and waves interact strongly with the heterogeneities and propagate along the multiple scattering trajectories, which are longer and much more complicated than the direct wave or simple reflected ones. Coda wave is an accumulation of all scattered, diffuse waves which are highly repeatable. Subtle changes in the medium are amplified by repeated sampling [17]. Therefore, the coda wave has a higher sensitivity to weak perturbations in the medium. The scientific fundamental of the CWI method is to extract information from two US signals recorded in different states as shown in Figure 1. It is easy to see that the first arrivals of two signals are almost identical; however, a slight waveform variation and time-domain perturbation are observed in the coda wave part (later arrivals).

The features extracted by the CWI method are relative velocity change (dV/V) and correlation coefficient (CC). In this method, the velocity change is considered to be dilation or compression in time by a factor $\alpha_{best}$. The most promising method to calculate $\alpha_{best}$ is the stretching method [31]. First, a reference signal $u_{u}\left(t\right)$ is chosen at an initial state, and then stretched by different factors α in a range $\left[\alpha_{min},\alpha_{max}\right]$ with a resolution of $10^{-5}$ or even $10^{-6}$. CC between the signal recorded in a new state $u_{p}\left(t\right)$ and all the stretched reference signals $u_{u}\left(t\left(1+\alpha \right)\right)$ will then be calculated. The α which maximizes the CC is chosen as the velocity change.

(1) $$CC\left(\alpha \right)=\frac{\int_{t-T}^{t+T}u_{u}\left(t^{\prime }\left(1+\alpha \right)\right)u_{p}\left(t^{\prime }\right)dt^{\prime }}{\sqrt{\int_{t-T}^{t+T}u_{u}^{2}\left(t^{\prime }\left(1++\alpha \right)\right)dt^{\prime }\int_{t-T}^{t+T}u_{p}^{2}\left(t^{\prime }\right)dt^{\prime }}}$$

**Figure 2:**
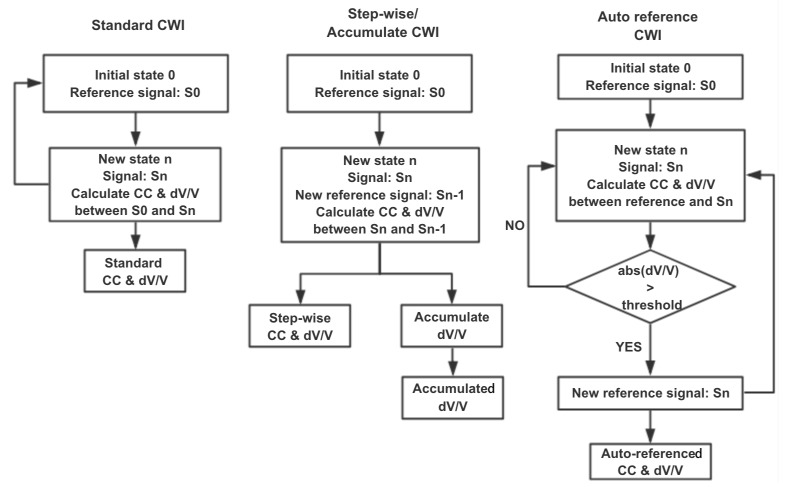
Algorithm of three CWI methods.

Regularly, the standard CWI method chooses a fixed reference to calculate the CWI properties. Nonetheless, when the changes in the medium reach a certain level, the waveform and/or the time-shift of the US signal in the new state could change substantially. Even though CC can still be useful as it presents the similarity of two signals, the dV/V is not meaningful anymore. In this case, step-wise CWI or auto reference CWI procedures, which changes the reference signal according to different situations, are proposed [32]. In the step-wise CWI method, a moving reference is used; CC and dV/V are calculated by the current signal and its previous signal. The step-wise dV/V could be accumulated to provide an overall change compared to the first reference signal. However step-wise CC cannot be multiplied as it is non-unique [32]. Damage such as crack openings might cause a velocity variation of more than 1% [27]. In this paper, when the absolute value of dV/V exceeds the threshold of 1%, the reference signal will be switched to the current US signal automatically; this is the so-called ‘auto reference’ CWI. This method limits the dV/V variation range to [–1, 1] which gives us a clearer view of velocity change when it is relatively small. The algorithms of these methods are shown in Figure 2.

## 3. Experimental Investigation

### 3.1. Transducer and Data Acquisition System

**Figure 3:**
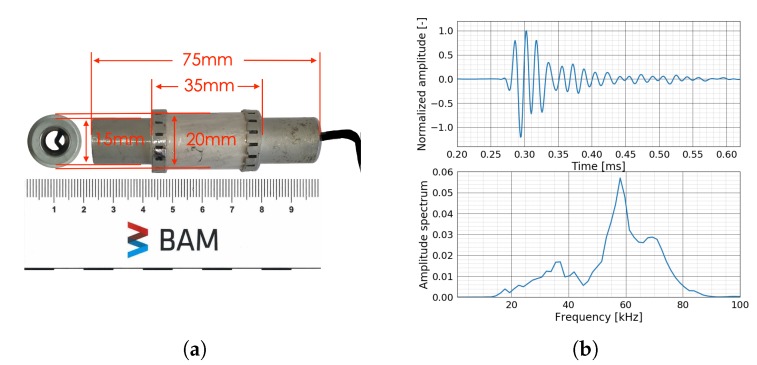
(**a**) Dimension of transducer SO807. (**b**) Response of transducer SO807 recorded in water (top) and the corresponding amplitude spectrum (bottom).

The ultrasonic sensor used in these experiments is ‘SO807’ which is a hollow piezoceramic US transducer designed by Acoustic Control Systems, Ltd. exclusively for BAM. It can be easily installed in the structure during the construction before casting [26,29]. Moreover, a special installation method is developed allowing its installation on existing structures [25,28]. As the transducer is installed inside the structure, the interior of the structure is better monitored and the influence of near-surface changes is reduced. SO807 can be used as both transmitter and receiver. Figure 3a shows the dimension of SO807 transducer. Two SO807 transducers were placed 40 cm away from each other in parallel in water. A short pulse of 60 kHz excited one transducer and the first arrival received by the other one is shown in Figure 3b. According to the amplitude spectrum, the central frequency peak is around 58 kHz, which leads to the interaction between waves and heterogeneities.

**Figure 4:**
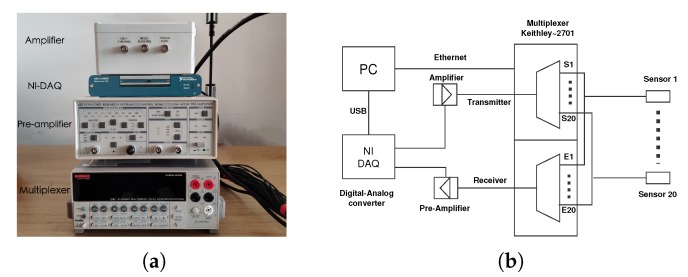
(**a**) Data acquisition devices (**b**) Diagram of the data acquisition system.

The data acquisition system (Figure 4) allows continuous monitoring with a maximum of twenty US transducers with a sampling frequency of 1 MHz. If necessary, the temperature can be also recorded for long-term monitoring. As the experiments described in this paper were performed in a laboratory environment, the influence of temperature was negligible. Transducer transmitter-receiver pairs can be controlled and configured easily in the control program. All the US data could be synchronised to the specified FTP server automatically, which allows remote measurement. The whole system includes:

- Amplifier to amplify the input signal of transmitter
- Pre-amplifier with analog filter to improve the signal recorded by receiver
- Digital-analog data acquisition module to convert signal
- Multiplexer with 20 channels to switch between different combinations of transducers
- PC with control software and data storage

### 3.2. Experimental Setup

#### 3.2.1. Test Specimen Design

**Figure 5:**
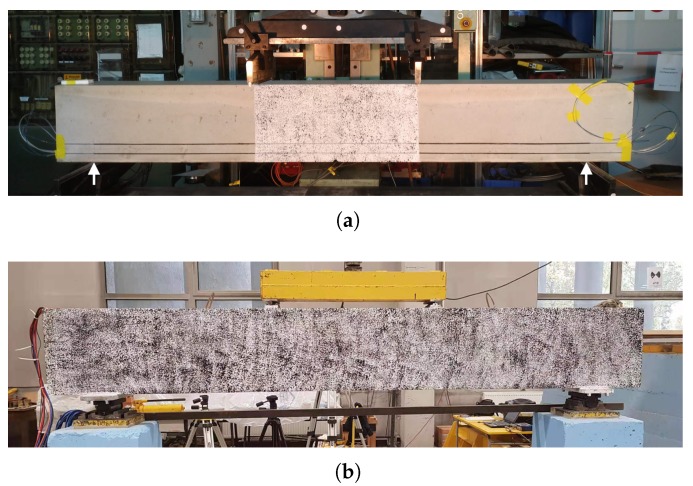
Front view of the beams and contract points position (**a**) At BAM and (**b**) at NEOSTRAIN.

**Figure 6:**
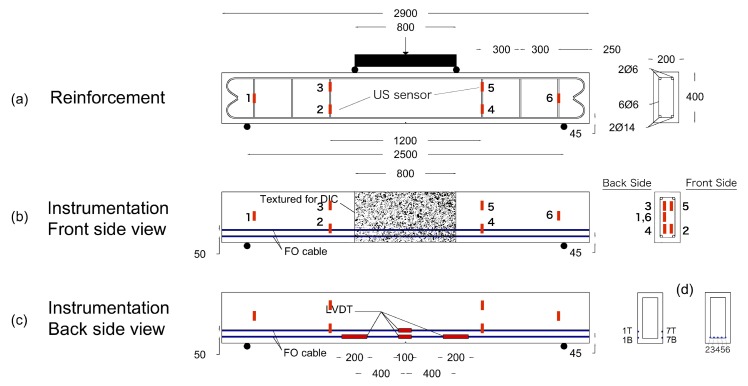
Dimensions of the beam, rebars distribution and sensors positions at BAM.

**Figure 7:**
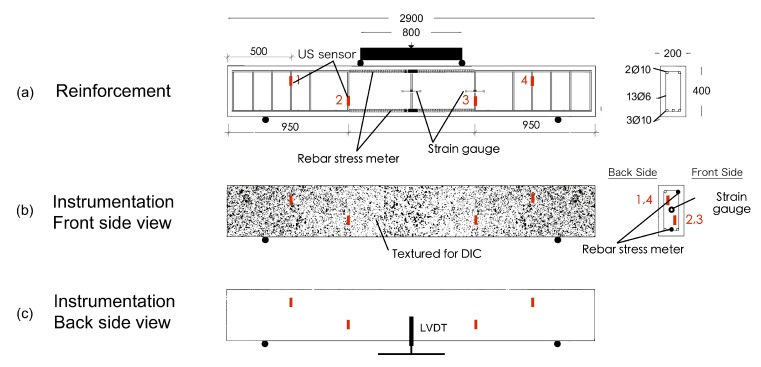
Dimensions of the beam, rebars distribution, and sensors positions at NEOSTRAIN.

Two similar RC specimens were designed to perform four-point bending tests at BAM and NEOSTRAIN. The dimension of the beams is 290 cm × 20 cm × 40 cm (length × width × height). The beam at BAM (Figure 5a and Figure 6) was reinforced with two ϕ 6 rebars in the compression zone and two ϕ 14 rebars in the tension zone, attached by six ϕ 6 stirrups with a spacing of 30 cm. The beam at NEOSTRAIN (Figure 5b and Figure 7) was reinforced with three ϕ 10 rebars in the tension zone and two ϕ 10 rebars in the compression zone, attached by thirteen ϕ 6 stirrups.

Multiple sensoring techniques are applied in the two bending test. The details of the techniques are listed in Table 1.

A limited number of US transducers was attached on the stirrups and installed inside both beams before casting. Two vibrating wire strain gauges and two rebar stress meters were installed in the beam at NEOSTRAIN to measure the strain variation in the beam and the rebar. As the quantity and position of all the cracks were not predictable, only four LVDT sensors were glued on the back surface of the beam at BAM to measure the Crack Opening Displacement (COD) for a few cracks on the surface and to double verify the FOS measurement. Strain changes and crack width inside the beam were also measured by FOS. Although FOS technique was not applied at NEOSTRAIN, DIC technique which covered the whole beam still provided a roughly COD and propagation map. Position and spacing between all the transducers are shown in Figure 6 and Figure 7.

#### 3.2.2. Loading Procedure

**Figure 8:**
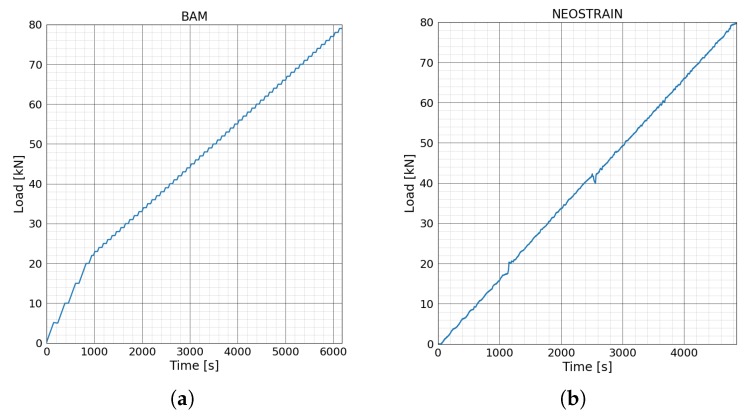
Loading steps of four points bending test at (**a**) BAM and at (**b**) NEOSTRAIN.

A step-wise loading procedure was performed for the test at BAM due to the low measurement frequency of the interrogator of FOS technique. In total, 64 load steps were performed. The load increased 5 kN/min and remained stable for one minute until 20 kN. For the following steps, the load increases 1 kN/min and remained stable for one more minute until reaching 80 kN as shown in Figure 8a. The loading procedure of the test at NEOSTRAIN was performed continuously with a increasing rate of 1 kN/min. Since the loading procedure at NEOSTRAIN was controlled manually, the load increased unexpectedly from 18 kN to 19.4 kN due to a mistake of the operator (Figure 8b).

## 4. Results

### 4.1. Crack Position

**Figure 9:**
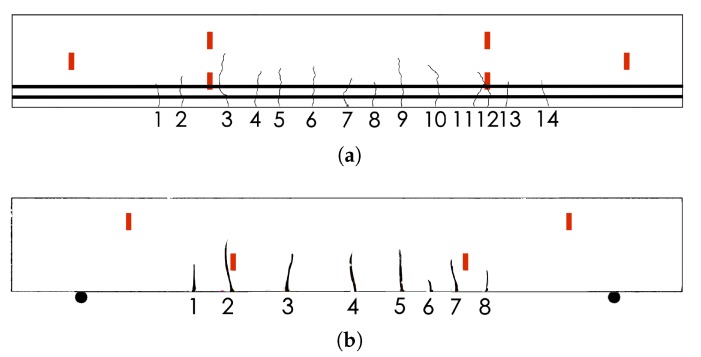
Cracks map of beam at (**a**) BAM and at (**b**) NEOSTRAIN.

At the end of the test, fourteen cracks appeared at the beam at BAM and eight cracks occurred in the beam at NEOSTRAIN. The locations of the cracks are presented in Figure 9. Cracks were mainly distributed between the two bottom US transducers. No crack was located in between the direct path of top level US transducers.

### 4.2. Deflection

**Figure 10:**
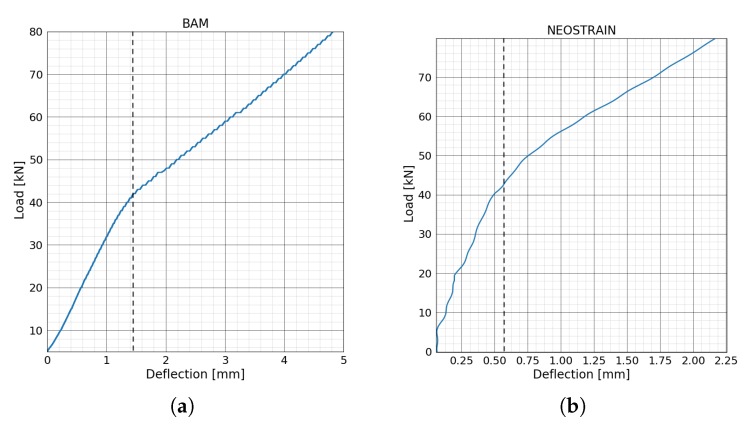
Load-deflection curve for bending test at (**a**) BAM and at (**b**) NEOSTRAIN.

The deflections of two beams during the four-point bending test are shown in Figure 10. Load-deflection curves for both beams lost their linearity at around 42 kN; the beams went into the plastic phase from the elastic phase, meaning that single or multiple cracks appeared. As mentioned in the previous section, the loading procedure at NEOSTRAIN was controlled manually; there was a small load fluctuation during the whole test.

### 4.3. Displacement and Strain

**Figure 11:**
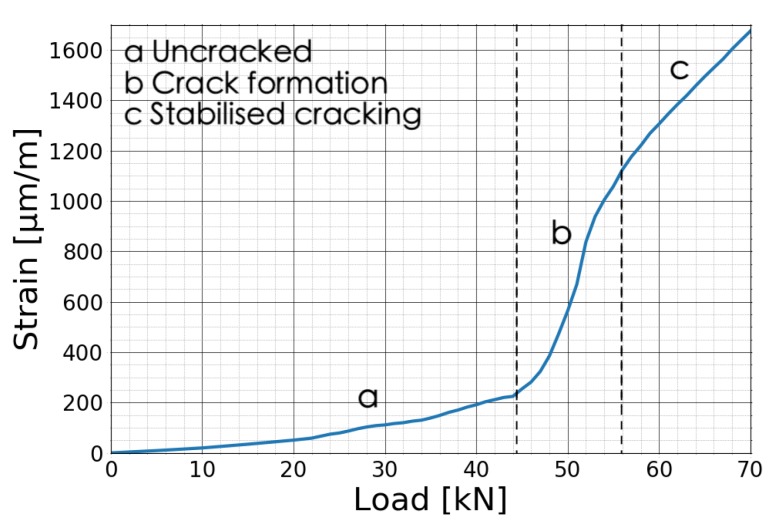
Force-strain curve of crack 5 measured by fiber line 7B.

The stress-strain relationship can distinguish between different stages of the crack opening: the uncracked stage, the crack formation stage and the stabilised cracking phase [33]. Figure 11 shows the three different stages of crack number 5 in the beam at BAM measured by fiber line 7 on the bottom in the front. The measured area remained uncracked until 45 kN, then the crack started to form and went into the stabilised cracking phase at 56 kN.

**Figure 12:**
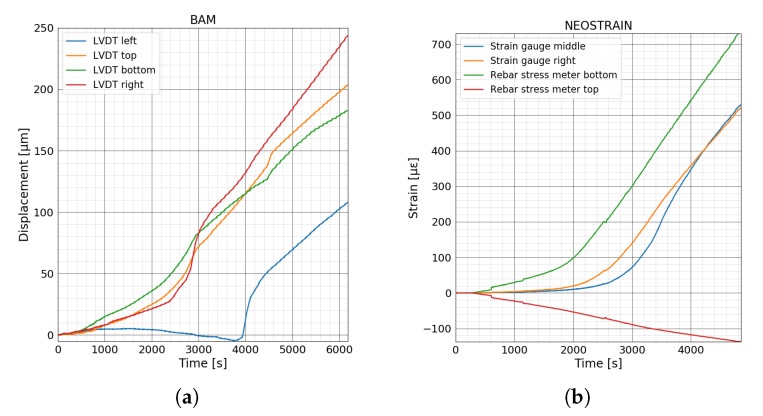
(**a**) Displacements measured by LVDT sensors at BAM. (**b**) Strain variation inside concrete and rebars measured by strain gauges and rebar stress meters at NEOSTRAIN.

**Figure 13:**
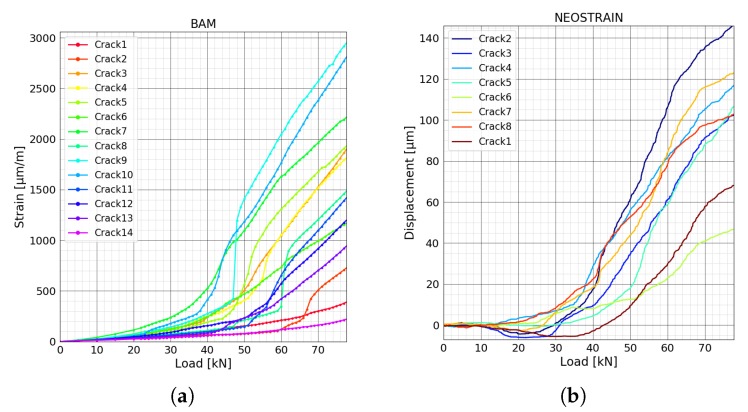
(**a**) Strain variation at position of all cracks measured by fiber line 7B at BAM. (**b**) Displacement at position of all cracks measured by DIC technique at NEOSTRAIN.

Figure 12a shows the COD of crack number 4, 7 and 10 measured by four LVDT sensors. The results are in accordance with the FOS measurement. A comparison of FOS technique, DIC, and LVDT in this experiment is presented in [34]. As fibers covered the whole beam and successfully detected all of the fourteen cracks in this experiment, the loads corresponding to the different stages of the crack opening are only determined by the force-strain curves measured by FOS (Figure 13a). Strain variations inside the beam and rebar at NEOSTRAIN are shown in Figure 12b. The top rebar was always under compression. The slope of two strain gauges started to change around 40 kN. The stress in the bottom rebar increased slowly until 32 kN and then increased linearly and rapidly after 35 kN.

Figure 13a shows the strain variation for all the crack positions measured by FOS during the bending test at BAM. Assuming that the strain variations are caused by the deformation discontinuity, COD could then be estimated [34]. This paper focuses only on the detection of the load level when cracks enter different phases, thus the calculation of COD is not necessary and not presented. The FOS technique was not implemented for the test at NEOSTRAIN; nonetheless, the approximate CODs of all the cracks were measured by the DIC technique, which covered the whole front surface. The DIC technique detected the COD by analysing the deformation of the surface of the beam which is caused by strain change [9]. Hence, different crack opening phases can also be determined by the stress-displacement curve Figure 13b. When the deformation is relatively small, the displacement measurement is strongly disturbed by the friction. This is the reason for the unusual negative displacement values at the beginning of the experiment.

The corresponding load to the moment when cracks enter different phases are listed in Table 2 and Table 3. As one can see, crack number 7 and 10 first appeared around 38 kN in the beam at BAM. Crack number 14 appeared the latest at 70 kN. In general, most of the cracks appeared after 42 kN matching well with the deflection measurement. Cracks 3, 4, 5 and 13 reached ‘stabilised cracking’ phase at around 56 kN along with crack numbers 6, 7, 8, 11 and 12 at around 60 kN. DIC technique showed that the first crack was located in the middle of the beam at NEOSTRAIN and appeared at around 37 kN. Almost all the cracks started to form at around 41 kN and left the formation phase at around 68 kN.

### 4.4. CWI Technique

**Figure 14:**
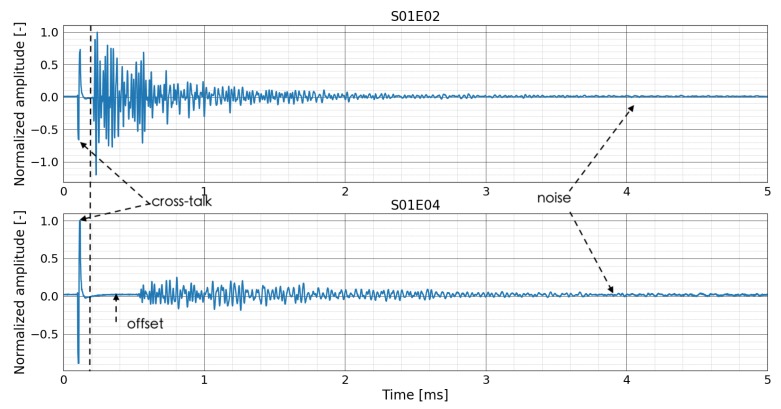
Correlation coefficient and relative velocity change of all the transducers combinations in test at BAM.

As an example, signals measured by transducer combinations S01E02 and S01E04 (SxxEyy: transmitter *xx* and receiver *yy* during the initial stage of the beam at NEOSTRAIN are plotted in Figure 14. The signals need to be pre-processed to filter the noise and to remove offset and cross-talk (more details can be found in [32]). As the distance between transducer number 1 and 4 is longer than that of transducer number 1 and 2, the signal received by transducer combination S01E04 has a longer time of flight and lower amplitude due to the attenuation of US wave in concrete.

#### 4.4.1. Test at BAM

**Figure 15:**
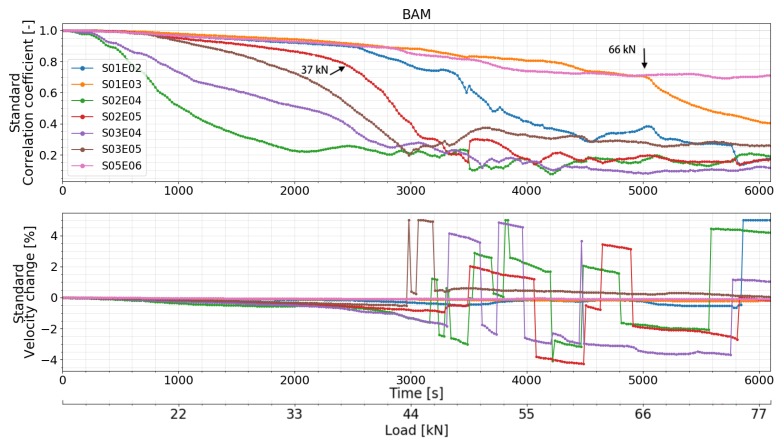
Correlation coefficient and relative velocity change of all the transducers combinations in test at BAM calculated by standard CWI.

**Figure 16:**
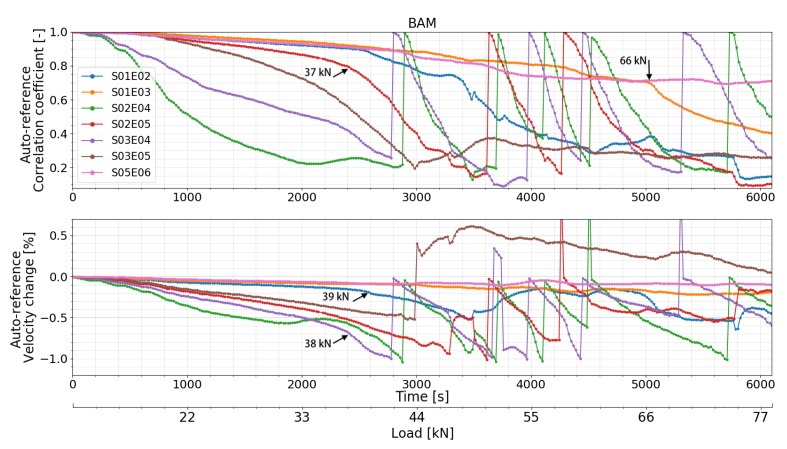
Correlation coefficient and relative velocity change of all the transducers combinations in test at BAM calculated by auto reference CWI.

**Figure 17:**
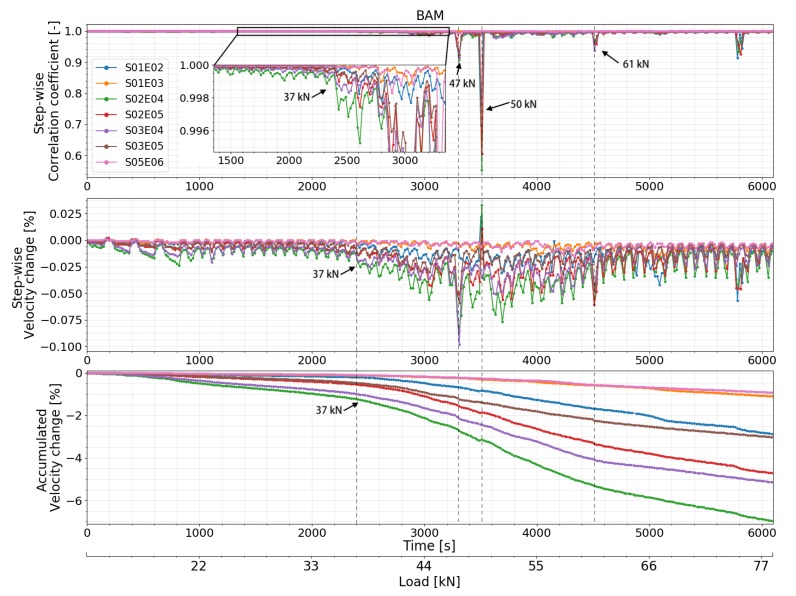
Correlation coefficient and relative velocity change of all the transducers combinations in test at BAM calculated by step-wise and accumulate CWI.

CC and dV/V of all the transducer combinations calculated by all the different CWI methods are presented in Figure 15 (standard CWI), Figure 16 (auto reference CWI), and Figure 17 (Step-wise CWI). Reference signals of all transducer combinations were recorded at the initial state of the beam before the start of the loading procedure. CC and dV/V were then calculated by normal CWI procedure. For the auto reference CWI, when the absolute value of dV/V exceed 1%, the damage in the structure reached a certain level. It is difficult to compare the following signals with the original reference signal. Thus, the current signal was chosen as a new reference signal until dV/V reached 1% again.

Unusual behaviors of transducer 04 were detected. The CC and dV/V of all the transducer combinations related to transducer 04 varied too much compared to other combinations which were located in the symmetrical positions of the beam. Signals recorded by S02E04 and S03E04 before the start of load test showed that the performance of transducer 04 was not so stable. However, the CC and dV/V related to transducer number 4 calculated by step-wise CWI are still helpful. Before the first cracks appeared, all the transducer combinations successfully detected the different loading steps, especially by step-wise CWI method. The CC and dV/V of transducer combination S02E05 lost their linearity (Figure 15 and Figure 16) at around 37 kN which indicates one or more very early stage crack(s) opening. This change was also detected by almost all the transducer combinations by step-wise CWI method (Figure 17), while by standard and auto reference methods, transducer combination S01E02 detected this change at 39 kN, as the crack is not in between its direct path area. The combinations of transducers S01E03 and S05E06 were located in the left and right corner on the top of the beam where there was no crack occurrence during the whole experiment. Nevertheless, they still detected the first crack(s) opening at 39 kN. This is very close to the moment when the first cracks started to form. Transducer combination S03E04 detected the first cracks at 38 kN. When the dV/V of transducer combination S03E04 reached –1% at 44 kN, a new reference was chosen automatically; furthermore, the continuous load was detected again. At 66 kN, CC measured by transducer combination S01E03 decreased rapidly. Using the step-wise CWI method, a significant change was detected at 47 kN (Figure 17) by all the combinations between transducer 02, 03, 04 and 05 which were located in the main stress change part of the beam; this indicated a higher level of damage in the beam. A second significant change was detected at 50 kN by all the transducer combinations related to transducer 02, which means the damage was close to transducer 02. According to the accumulated CWI, CC, and dV/V of transducer combinations S01E03 and S02E05 varied the least as they were not located in the damage area.

#### 4.4.2. Test at NEOSTRAIN

**Figure 18:**
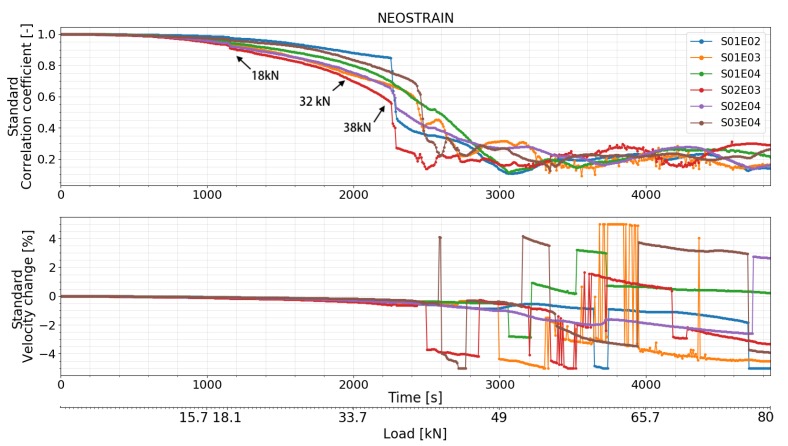
Correlation coefficient and relative velocity change of all the transducers combinations in test at NEOSTRAIN calculated by standard CWI.

**Figure 19:**
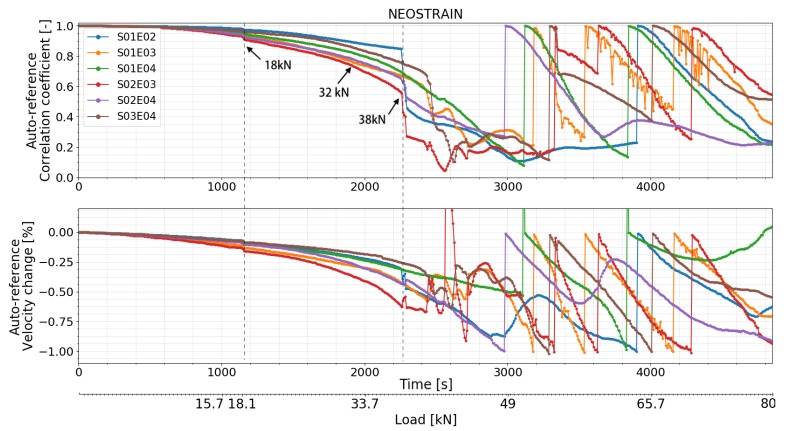
Correlation coefficient and relative velocity change of all the transducers combinations in test at NEOSTRAIN calculated by auto reference CWI.

**Figure 20:**
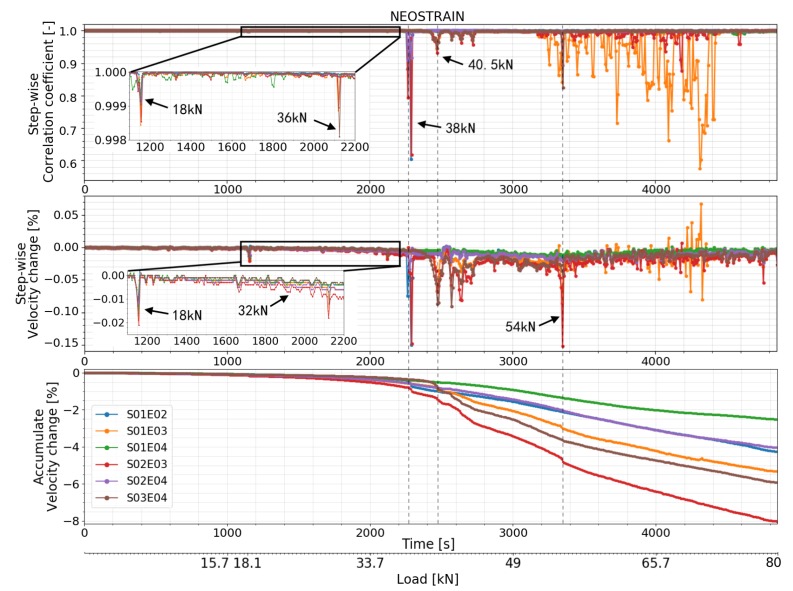
Correlation coefficient and relative velocity change of all the transducers combinations in test at NEOSTRAIN calculated by step-wise and accumulate CWI.

As shown in Figure 18, Figure 19 and Figure 20, small variations in CC and dV/V were observed at 18 kN which corresponded to the misoperation mentioned in the ‘loading procedure’ section. Both features of all transducer combinations then decreased smoothly. CC and dV/V of transducer combination S02E03 decreased with a higher rate at 32 kN. A small change was detected by transducer combinations S01E03, S02E03, and S03E04 at 36 kN by step-wise CWI method. A second strong change can be seen in the CC and dV/V of transducer combinations S01E02, S02E04, and S02E03 at 38 kN. CC and dV/V of all transducer combinations related to transducer number 3 (S01E03, S02E03, and S03E04) started to change irregularly from 40.5 kN, meaning that the multiple cracks occurred near transducer number 3. This is close to the moment when the beam entered plastic phase. CC and dV/V measured by the top level transducer combination S01E03 had their first sudden change at 54 kN. This means either cracks affected the direct path between them or the global damage in the beam reached a certain level.

## 5. Discussion

According to the results of both experiments, the CWI method shows a high sensitivity to stress change in the structure. In the uncracked phase, when the tensile stress increases, relative velocity decreases linearly. When cracks started to form, the linearity was broken.

### 5.1. Test at BAM

In a real case, the exact locations of cracks are normally unpredictable, thus LDVT sensors were used only as an auxiliary tool. The DIC technique is easy to implement; however, to compare with FOS, the measuring range and accuracy are relatively low. The LVDT and DIC results proved the reliability of the FOS measurement. The crack opening and location are determined by FOS technique. The first crack(s) opening was detected at 37 kN by transducer combination S02E05 and at 38 kN by transducer combination S03E04, the paths of which covered the main middle part of the beam. FOS detected two crack openings (crack number 7 and 10) at 38 kN. CWI method detected the cracks earlier than all other techniques; however, it has difficulties to quantify them precisely. FOS detected formation of crack number 3 at 40 kN which leads to the significant decrease of CC and dV/V measured by transducer combination S01E02 at 39 kN as crack number 3 was located very close to transducer number 2. Transducer combinations S01E03 and S05E06 detected these cracks at 39 kN during their ‘crack formation’ phase. It is notable that these cracks were not close to any of these transducers and they were not even located in between the direct path area of these transducers. The dV/V measured by transducer S03E04 reached −1% at 44 kN; the authors assume that the damage in the beam exceeded a certain level. The CC of transducer combination S01E03 decreased rapidly at 66 kN, which is caused by the appearance of crack number 2.

### 5.2. Test at NEOSTRAIN

The FOS technique was not used in this experiment. Nonetheless, the DIC technique provided a rough COD measurement and clear cracks map. CC and dV/V of all transducer combinations varied suddenly at 18 kN due to a misoperation during the load procedure. This proves again the high sensitivity of CWI method to stress variation. A change was detected by CC and dV/V of transducer combination S02E03 at 32 kN, which is the moment when stress in the bottom rebar lost its linearity. The authors assume it as a sign before the first crack appeared. According to the COD measured by DIC, the first crack (crack 4) appeared at 37 kN and was located in the middle of the beam. All the transducer combinations related to transducer number 2 detected a change in dV/V and CC, which indicates the creation of crack number 2. According to DIC, all the other cracks beside crack number 6 started to form at around 41 kN. CC and dV/V measured by transducer combinations S01E03, S02E03, and S03E04 started to fluctuate at 40.5 kN because of crack number 7 and 8, which surrounded transducer number 3. During the ‘crack formation’ phase, crack number 2, 4, 7, and 8 had bigger COD than the others (Figure 13).

## 6. Conclusions and Remarks

In this study, three CWI algorithms show their feasibility and reliability. Each of them has both advantages and disadvantages. The standard CWI method requires the shortest processing time as it stretched only once the first reference signal. It shows the continuous variation compared to the initial state. However, when cracks appear and the damage in the structure reaches a certain level, it is hard to extract information from both CC and dV/V. Thus, the auto reference CWI method is proposed when the velocity change reached a threshold and a new reference is chosen. This method reduces the dV/V variation range and provides a clearer view. The step-wise CWI method needs a heavier processing procedure as the reference changes all the time; therefore, all the signals need to be stretched. To accelerate the calculation, a lower resolution could be chosen to calculate dV/V as the change between two adjacent signals are not that big. Nonetheless, step-wise CWI shows the clearest and most precise local variation. The dV/V could be accumulated to observe the global variation, relative to the beginning of experiment. The unusual sudden changes observed by standard or auto reference CWI are successfully eliminated. This is the so-called accumulated CWI.

The CWI method shows a very high sensitivity to detect stress changes in the specimens. The increasing bending tensile stress leads to a decrease in relative velocity change. Its capability to detect early stage damage in reinforced concrete is also proven. CWI detects cracks earlier than all the other standard traditional sensors, even though the cracks are not in the direct path area between two transducers. This shows the independence of CWI technique from the location of the structural flaw. Although CWI technique could not detect the quantity and location of all the cracks as precisely as FOS and DIC do, an approximate location and damage level of crack(s) can be still inferred in a very early stage. Moreover, as compared with FOS technique, the cost of data acquisition system is significantly lower and the embedded US transducers have higher damage resistance than FOS. The two specimens were monitored by limited numbers of US transducers continuously during the whole bending test. In practical applications, the authors suggest that the use of only two US transducers is enough to monitor a three-meter beam. Due to the easy installation of SO807 transducer and the small size of the US data, which allows remote monitoring, CWI method combined with embedded US transducers will definitely have a bright future for long-term monitoring in SHM.

## Figures and Tables

**Table 1:** Techniques and sensors used in two tests.

Technique	BAM	NEOSTRAIN
US transducer	6 SO807	4 SO807
Strain gauge	0	2 vibrating wire strain gauges
		2 rebar stress meters
LVDT	4	1
Fiber optic sensor	9 fibers	0
DIC	80 cm × 40 cm	290 cm × 40 cm

**Table 2:** Moment when crack enters ‘crack formation’ phase and ‘stabilised cracking’ phase at BAM.

Phase	Crack 1	Crack 2	Crack 3	Crack 4	Crack 5	Crack 6	Crack 7
Crack formation	67 kN	60 kN	40 kN	45 kN	45 kN	40 kN	38 kN
Stabilised cracking	None	68 kN	56 kN	56 kN	56 kN	61 kN	61 kN
**Phase**	**Crack 8**	**Crack 9**	**Crack 10**	**Crack 11**	**Crack 12**	**Crack 13**	**Crack 14**
Crack formation	48 kN	47 kN	38 kN	51 kN	51 kN	42 kN	70 kN
Stabilised cracking	60 kN	50 kN	46 kN	60 kN	60 kN	58 kN	None

**Table 3:** Moment when crack enters ‘crack formation’ phase and ‘stabilised cracking’ phase at NEOSTRAIN.

Phase	Crack 1	Crack 2	Crack 3	Crack 4	Crack 5	Crack 6	Crack 7	Crack 8
Crack formation	41 kN	40 kN	41 kN	37 kN	41 kN	53 kN	41 kN	41 kN
Stabilised cracking	70 kN	61 kN	68 kN	42 kN	58 kN	68 kN	68 kN	68 kN

## Abbreviations

The following abbreviations are used in this manuscript:

BAM	Bundesanstalt für Materialforschung und-prüfung
RC	reinforced concrete
SHM	Structural Health Monitoring
NDT	Non-Destructive Testing
CWI	Coda Wave Interferometry
FOS	Fiber Optics Sensor
AE	Acoustic Emission
UPV	Ultra Pulse Velocity
CC	Correlation Coefficient
US	Ultrasonic
DIC	Digital Image Correlation
LVDT	Linear Variable Differential Transformer
COD	Crack Opening Displacement
